# Temperature and Strain Rate Related Deformation Behavior of UHMWPE Fiber-Reinforced Composites

**DOI:** 10.3390/polym16091250

**Published:** 2024-04-30

**Authors:** Chenhong Yi, Jianhui Xu, Lizhi Tian, Chun Zhang

**Affiliations:** 1 Institute of Fluid Physics, China Academy of Engineering Physics, Mianyang 621000, China; yichenhong1975@caep.cn (C.Y.); xujianhui@mail.nwpu.edu.cn (J.X.); 2 School of Aeronautics, Northwestern Polytechnical University, Xi’an 710072, China; 3 Institute of Aeronautical Composite Structures, Northwestern Polytechnical University, Xi’an 710072, China; 4 AVIC Jincheng Nanjing Engineering Institute of Aircraft Systems, Nanjing 211106, China

**Keywords:** UHMWPE fiber-reinforced composites, viscoelastic, constitutive model, tensile test, temperature and strain rate

## Abstract

As they possess the qualities of high specific strength, high specific modulus, high specific energy absorption, and excellent designability, ultra-high molecular weight polyethylene (UHMWPE) fiber-reinforced composites have gradually replaced traditional materials such as ceramics and steel plates as the main ballistic protection materials. Using an improved test method, the uniaxial tensile tests of UHMWPE fiber-reinforced composites at two strain rates of 10^−4^ s^−1^ and 10^−2^ s^−1^ and a temperature range from −20 °C to 80 °C are carried out to study the effects of strain rate and temperature on the tensile behavior of UHMWPE fiber-reinforced composites. The experimental results indicate that the tensile responses exhibit nonlinear characteristics and the sensitivity of strain rate and temperature. The yield strength and modulus decrease with increasing temperature and increase with the increase in strain rate. A phenomenological viscoelastic constitutive model composed of a nonlinear spring and a nonlinear Maxwell element is proposed to characterize the temperature and strain rate dependent deformation behavior of UHMWPE fiber-reinforced composites before yielding. The results show that the model can accurately predict the tensile nonlinear viscoelastic responses of UHMWPE fiber-reinforced composites before yielding over a wide temperature range under quasi-static loading.

## 1. Introduction

Ultra-high molecular weight polyethylene (UHMWPE) is a kind of thermoplastic engineering plastic with linear structure and excellent comprehensive properties. The high-strength fibers based on this material have excellent mechanical properties, chemical medium resistance, impact resistance, moisture and corrosion resistance, and can be combined with different matrixes to make various types of composites [1,2,3,4]. UHMWPE fiber-reinforced composites are widely used in military protection, aerospace, sports medicine, and other fields, due to their various excellent characteristics [5,6,7,8,9]. Among them, ballistic protection is the most important application of UHMWPE fiber-reinforced composites, and their light weight, high strength, and impact resistance properties promote ballistic protection equipment to gradually develop in the direction of high efficiency, light weight, and integration [10,11]. Unlike traditional composite materials, such as carbon fiber-reinforced resin composites, the role of the matrix in these materials is not to transfer the load to the fibers, but to allow the fibers to engage with the projectile during high-speed impact. Therefore, the interface bonding between fibers and matrix does not play any important role in the load, and the matrix used is essentially soft and tough to achieve better ballistic performance. The mechanical response of UHMWPE fiber-reinforced composites under ballistic loading is the result of the joint action of mechanical behaviors such as tensile, compression, shear, and interlayer delamination [12,13,14,15,16], among which in-plane tensile deformation energy absorption is the most fundamental property of UHMWPE fiber-reinforced composites in the design of ballistic protection materials [16,17,18,19,20]. Therefore, the study of in-plane tensile behavior of UHMWPE fiber-reinforced composites is of great significance to reveal their intrinsic mechanical responses under ballistic loading.

Many scholars have carried out numerous experimental and theoretical works on the tensile behavior of UHMWPE fiber-reinforced composites. UHMWPE fiber-reinforced composite laminates with orthogonal unidirectional (UD) structure have no interweaving between the layers of fibers, low matrix content, and the existence of gaps inside the material. The interlaminar shear strength is very low compared to the tensile strength [21,22,23], which leads to the tendency of delamination slippage in tensile property tests of UHMWPE laminates and affect the reliability of test results. In this regard, numerous scholars have carried out a lot of fruitful work on the tensile property tests of UHMWPE fiber-reinforced composites by improving the test methods. Kartikeya et al. [24] used five test methods to conduct tensile tests on eight different grades of UHMWPE fiber fabrics and explored the advantages and disadvantages of each method. The tensile properties of UHMWPE fiber-reinforced composites were evaluated by using a UHMWPE single-ply tensile test instead of a UHMWPE laminate tensile test. A set of simple fixtures was developed to overcome the problem of delamination slippage during UHMWPE single-ply tensile test, and the test results showed that the tensile strength was close to the theoretical value of the tested material. Russell et al. [25] investigated the tensile response of UHMWPE fiber-reinforced composite laminates in the low strain rate (10^−4^–10^−2^ s^−1^) range by bolting through the bony specimen clamping end, and also conducted tensile tests on UHMWPE fiber bundles in the wide strain rate range. The test results indicated that the tensile strength of fiber bundles was 20% higher than that of laminates, and the tensile response of fiber bundles in the high strain rate (10^−1^–10^3^ s^−1^) range was insensitive to the strain rate.

Meanwhile, based on the tensile tests of UHMWPE fiber-reinforced composites, scholars have characterized the constitutive relationship of the material. Levi-Sasson et al. [26] designed a dog bone tensile specimen and fixed it with screws to a test fixture and performed tensile tests in the low strain rate range, based on the Ramberg–Osgood (RO) model, the constitutive relationship of UHMWPE fiber-reinforced composite laminates under tensile loading was investigated. Torsten et al. [27] conducted a detailed experimental characterization of the anisotropic directional strength under quasi-static conditions and the impact behavior of a UHMWPE fiber-reinforced composite laminate (Dyneema HB26). The study proposed a nonlinear constitutive material model of Dyneema HB26, which can describe the orthotropic anisotropic elastic and plastic-hardening behavior of the material and characterize the impact behavior. The model can implement the simulation of the material impact process at high speed by a finite element program. However, the model is based on the classical laminate theory, and the model is complex with many parameters, which makes the implementation process quite difficult.

The mechanical response of UHMWPE fiber-reinforced composites is influenced by many factors, including strain rate, temperature, thickness, moisture absorption, and molding process [28]. Therefore, some scholars have carried out research on these factors. Kromm et al. [29] investigated the tensile and creep properties of UHMWPE fibers under different temperature and strain rate conditions. It was found that Young’s modulus and tensile strength were less dependent on strain rate due to the fact that UHMWPE fibers were highly oriented and highly crystalline after being fabricated by the hot stretching method, which reduced the viscous response during fiber stretching. Meanwhile, the mechanical properties of UHMWPE fibers were greatly influenced by temperature. The main mechanical properties of UHMWPE fibers were determined by tensile and creep tests at different temperatures and loading conditions, and a constitutive model related to creep behavior was developed. Chen et al. [30] performed uniaxial tensile tests on UHMWPE fiber laminates of different thicknesses at quasi-static and high strain rate loading to investigate the effects of thickness and strain rate on the tensile properties and failure mechanisms of UHMWPE fiber laminates. The results showed that the influence of thickness on the quasi-static strength and modulus of UHMWPE fiber laminates was not significant, but affected the failure elongation. The tensile strength and elastic modulus of UHMWPE fiber laminates were sensitive to the strain rate. Under quasi-static and low strain rate loading, fiber pullout of UHMWPE fiber laminates was the main failure mode. Under high strain rate loading, the failure mode of the specimen was uniform fracture perpendicular to the direction of tensile load. The effect of the manufacturing process on the dynamic properties of UHMWPE composites (Dyneema HB26) was investigated for the first time by Torsten et al. [31]. The hot pressing parameters of temperature and pressure had a significant effect on the mechanical properties of the material. The impact resistance and shock wave properties of the UHMWPE composites consolidated under three different pressures were characterized. For UHMWPE composites, higher consolidation pressure would obtain better ballistic performance. Based on these results, an analytical method describing the equation of state as a function of consolidation pressure was proposed, which was able to truly predict the impact behavior of UHMWPE composites. Chouhan et al. [32] investigated the effect of hygroscopicity on the high strain rate compression property of UHMWPE composites along the thickness direction. The dynamic compression performance was studied using the split Hopkinson compression bar. The test results showed that the high strain rate compressive mechanical properties of the composites decreased after moisture absorption; the materials did not produce damage under low strain rate loading, and higher loading rate would lead to macro damage.

It is evident from the relevant literature that there is no uniform standard test method for testing the tensile properties of UHMWPE fiber-reinforced composites, and there are differences in the reported data. Researchers have explored many test methods to carry out the study of tensile properties of UHMWPE fiber-reinforced composites, but each method has certain limitations. Therefore, in this study, the tensile test method for UHMWPE laminates was explored and three specimens of different specifications were designed, and the final established method gave realistic and reliable results without delamination or slippage.

The experimental method studied in this article provides a simple and reliable reference for the mechanical testing of ultra-high molecular weight polyethylene fiber-reinforced composite materials in the future. Using this method, the quasi-static tensile tests of UHMWPE fiber-reinforced composites were carried out to study their tensile behaviors under different temperatures and strain rates, and a constitutive model was established to accurately characterize the mechanical response before yielding, with a view to providing data support and theoretical guidance for subsequent research on the ballistic performance of UHMWPE fiber-reinforced composites.

## 2. Materials and Experiment

### 2.1. Materials

**Figure 1:**
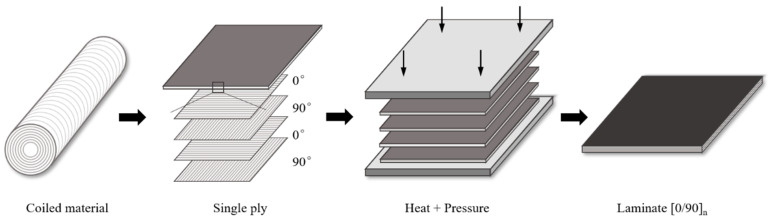
Preparation process of UHMWPE fiber-reinforced composite laminate.

The raw material used in this study is UHMWPE fiber-reinforced composite non-weft cloth, the material brand is AT40H120, the matrix is polyurethane, and the material specifications are shown in Table 1, which come from Ningbo Shuxian Aerospace Composite Material Co., LTD, Ningbo, Zhejiang Province, China. The non-weft cloth was cut into a suitable size by using the computer cutting machine, stacked according to [0/90]_n_, put into the mold for heating and pressure curing, heat preservation, and cooling to make UHMWPE fiber-reinforced composite laminate [25]. The preparation process is shown in Figure 1. The specific process is as follows:

- Preparation of tooling. Clean the molding fixture with ethyl acetate until the surface is free of oil stains, rust, and other pollutants. Brush the mold release agent 2–3 times horizontally and cross on the surface of the mold cavity.
- Material preparation. Place pre-impregnated materials in a dust-free room at 23 ± 5 °C for thawing 12 h in advance; according to the size and thickness of the laminated board, use a cutting machine to cut the prepreg. During the cutting process, be careful to avoid contaminating the prepreg. Lay the prepreg into the mold cavity of the molding fixture according to the laying angle of the laminated board.
- Equipment preparation. Set the 500T hot press machine to 200 °C for early heating; only after the heating plate temperature reaches 200 °C can the specimen be heated and cured.
- Curing of laminated boards. The curing temperature is 160 ± 10 °C, the pressure is 5–6 MPa, and the pressurization time is 30–40 min. Arrange 2–4 thermocouples at the edge of the molding fixture to monitor the fixture temperature. Calculate the pressurization time after the thermocouple temperature reaches 160 °C.
- Sample processing. After cooling the laminated board to room temperature, open the mold. After the mold is opened, use a CNC ultra-high pressure water cutting machine to roughly process rectangular test pieces. Then, bond and fix glass fiber reinforcement plates at both ends of the test pieces, and carry out precision machining again using a CNC ultra-high pressure water cutting machine.

### 2.2. Quasi-Static Tensile Test of Composite Materials

**Figure 2:**
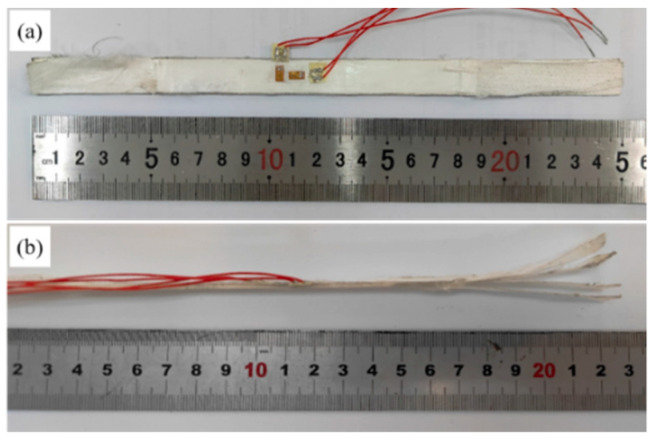
Rectangular specimen after stretching from (**a**) top view and (**b**) side view.

First of all, the uniaxial tensile tests were conducted with reference to ASTM D3039/D3039M-17 [33], and the direction is 0 degrees, as shown in Figure 1. Based on this point, we designed a rectangular tensile specimen with a length of 250 mm and a width of 15 mm and conducted uniaxial tensile tests on the specimen using a standard hydraulic fixture. Two orthogonal strain gauges were pasted on both sides of the specimen to measure the modulus and Poisson’s ratio of the composite material. In the early stage of the tensile test, as shown in Figure 2, it was found that the clamping part of the specimen was delaminated and slipped, and the test did not achieve the expected purpose.

**Figure 3:**
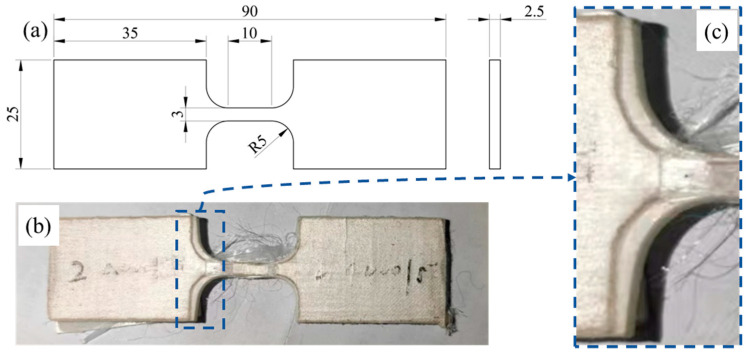
Dog-bone-shaped specimen of initial design; (**a**) specimen dimensions, (**b**) after tensile test, (**c**) delamination.

Secondly, we designed a dog-bone-shaped specimen with reference to the ISO 527-4 and previous studies on UHMWPE fiber-reinforced composites [24,25,30,34]. The specimen was cut into shape by water jet, the dimensions of the specimen are shown in Figure 3a; the specimen thickness is 2.5 mm, and the gauge length, width, and fillet radius of the specimen are 10 mm, 3 mm, and 5 mm, respectively. The width of the gripping portion is 25 mm, and the total length of the specimen is 90 mm.

Although the reference standards have been adjusted, due to the weak interlayer bonding performance of UHMWPE fiber-reinforced composite materials, as shown in Figure 3a, the modified quasi-static tensile specimen will not provide sufficient interlayer friction due to the small size of the dog-bone-shaped specimen’s clamping end, resulting in internal delamination and detachment of the specimen at the clamping end. The morphology and layering of the tensile test are shown in Figure 3b,c.

**Figure 4:**
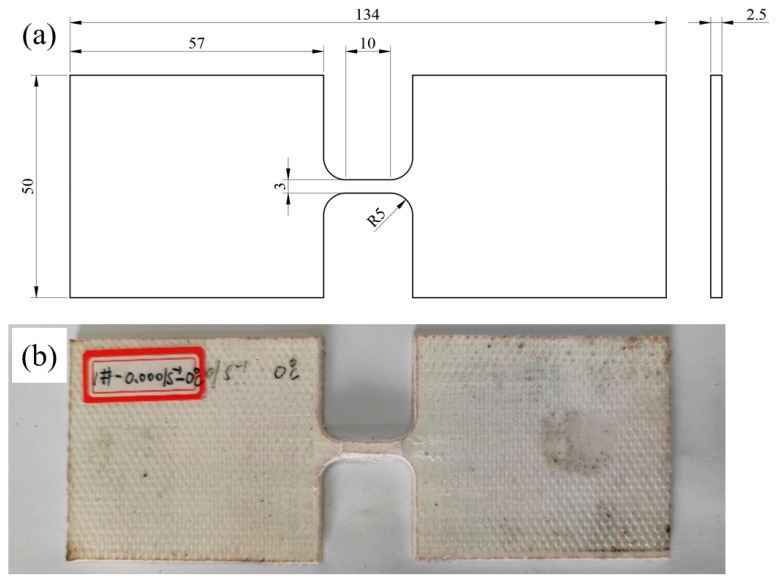
Improved dog-bone-shaped specimen; (**a**) specimen dimensions, (**b**) after tensile test.

To solve the above problems, it is necessary to increase the internal friction force of the test piece rather than the friction coefficient (which remains constant), which is used to break the gauge length section of the test piece. The dimensions of the third designed sample are shown in Figure 4a. Specifically, the size of the gauge length remains unchanged, the width of the clamping part is 50 mm, and the total length of the specimen is 134 mm. The photo of the tensile specimen is shown in Figure 4b.

To summarize, this article starts with selecting ASTM D 3039 [33] as a reference. Because UHMWPE fiber-reinforced composites cannot provide sufficient frictional force between layers to provide tensile load for the gauge range. Then, the ISO527-4 was selected. And finally, a sufficiently large clamping end specimen with a dog-bone shape was used to study its constitutive relationship.

All experiments were conducted on the DDL-100 electronic universal testing machine (Sinotest Equipment Co., Ltd. Changchun City, Jinpin Province, China), using hydraulic fixtures to ensure sufficient clamping force to avoid sliding of the specimen during loading. Install the temperature box on the testing machine and set the average heating rate to 3 °C/min. To obtain a uniform temperature distribution, keep the sample at the test temperature for 30 min before the start of the experiment.

Quasi-static tensile mechanical tests were conducted at five temperatures (−20 °C, 0 °C, 20 °C, 50 °C, 80 °C) and two strain rates (10^−4^ s^−1^, 10^−2^ s^−1^). At least three sets of tests should be conducted for each testing state to ensure the accuracy, reliability, and repeatability of the test results. The constant engineering strain rates were controlled by setting the loading speed of the testing machine.

Due to (a) difficulty in using an extensometer on a sample with a gauge length of 10 mm, (b) high- and low-temperature conditions during the test, (c) high elongation (generally exceeding 20%, the maximum exceeding 140%), and (d) low absolute load during the test (not exceeding 6.8 kN load, far less than the stiffness of the steel frame of the testing machine), we used the displacement of the testing machine’s cross head to replace the deformation of the test piece, and then calculated the test results such as strain and elongation. Additionally, Kromm [29] verified that the relative error between the measured displacement and the elongation of the specimen is within a reasonable range. The engineering stress and engineering strain are represented as follows:

(1) $$\sigma_{E}=\frac{F}{A}$$

(2) $$\varepsilon_{E}=\frac{\Delta L}{L_{0}}$$

where *F* is the force, N; *A* is the original cross-sectional area of specimen, mm^2^; Δ*L* is cross head displacement, mm; *L*_0_ is the original gauge length of specimen, mm; $\sigma_{E}$ is engineering stress, MPa; $\varepsilon_{E}$ is engineering strain. Assuming that the volume of the specimen remains unchanged before and after deformation, the true stress and the true strain can be expressed as follows:

(3) $$\sigma_{T}=\sigma_{E}(1+\varepsilon_{E})$$

(4) $$\varepsilon_{T}=\ln(1+\varepsilon_{E})$$

where $\sigma_{T}$ is true stress, MPa; $\varepsilon_{T}$ is true strain.

## 3. Results and Discussion

### 3.1. Tension Responses of UHMWPE Fiber-reinforced Composites

**Figure 5:**
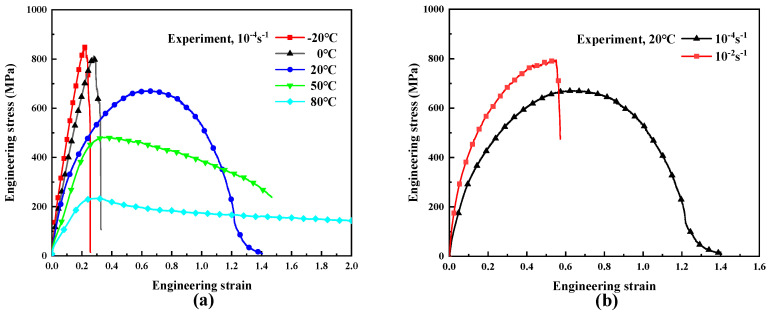
Uniaxial tensile engineering stress-strain curves of UHMWPE fiber-reinforced composites. (**a**) The different temperatures (−20 °C, 0 °C, 20 °C, 50 °C, 80 °C); (**b**) the different strain rates (10^−4^ s^−1^, 10^−2^ s^−1^).

Figure 5 shows the tensile engineering stress–strain curves of UHMWPE fiber-reinforced composites at different temperatures and strain rates. It can be seen that the tensile responses of UHMWPE fiber-reinforced composites are nonlinear and have a great relationship with the temperature and the strain rate. The elastic modulus decreases with increasing temperature and increases with increasing strain rate. Figure 5a shows that the tensile response of the material exhibits stronger linear viscoelastic behavior in the range of −20–0 °C, and the nonlinearity of the material is more pronounced in the range of 0–80 °C, which is attributed to the fact that molecular chains move slowly and require more energy to overcome free energy barriers at low temperatures [35]. As the temperature decreases, the toughness of the material decreases, showing a brittle fracture behavior. As the temperature increases, the yielding phenomenon of the material becomes more obvious, the yield stress and tensile strength decrease, and the fracture strain increases. Herein, yield stress is defined as the point on the stress–strain curve where the first increase in strain occurs without an increase in stress (ISO 527) [34]. Figure 5b demonstrates that the tensile responses of the UHMWPE fiber-reinforced composites exhibit obvious strain rate sensitivity, and similar phenomena can be observed at several other test temperatures.

### 3.2. Constitutive Model of Nonlinear Viscoelastic Responses

**Figure 6:**
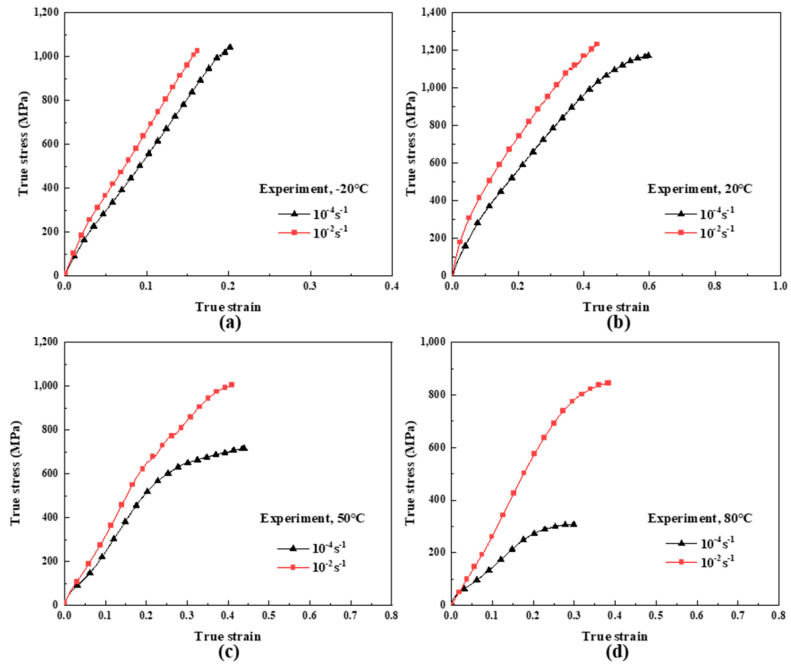
Tensile true stress-strain curves of UHMWPE fiber-reinforced composites before yielding. (**a**) −20 °C; (**b**) 20 °C; (**c**) 50 °C; (**d**) 80 °C.

**Figure 7:**
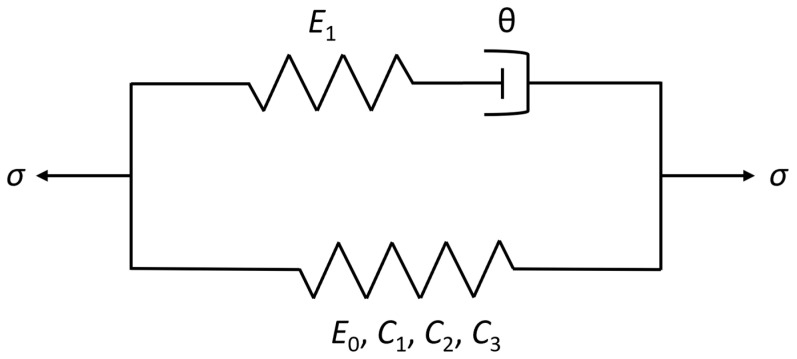
Nonlinear viscoelastic constitutive model.

Constitutive models describe the material responses to different mechanical and/or thermal loading conditions, which provide the stress–strain relations to formulate the governing equations, together with the conservation laws and kinematic relations. As shown in Figure 6a–d, the tensile behavior of UHMWPE fiber-reinforced composites before yielding exhibits nonlinear viscoelasticity and has an obvious temperature and strain rate dependence. Therefore, a phenomenological nonlinear viscoelastic constitutive model considering both temperature and strain rate is proposed to describe and predict the stress–strain relationship of the materials before yielding at different temperatures and strain rates. The schematic diagram of the model is shown in Figure 7. The model is composed of a quartic nonlinear spring and a nonlinear Maxwell element. The nonlinear Maxwell element consists of a linear spring and a nonlinear buffer, in which the relaxation time of the nonlinear buffer is not a constant and is defined as a function of strain rate to characterize the viscous responses of UHMWPE fiber-reinforced composites at different strain rates [36]. The equation expression of the constitutive model is as follows:

(5) $$\sigma =E_{0}\varepsilon +C_{1}\varepsilon^{2}+C_{2}\varepsilon^{3}+C_{3}\varepsilon^{4}+E_{1}\int_{0}^{t}\dot{\varepsilon }(\tau )\exp(-\frac{t-\tau }{\theta })d\tau$$

(6) $$\theta =\theta_{0}{\left(\frac{\dot{\varepsilon }}{{\dot{\varepsilon }}_{0}}\right)}^{-\beta }$$

where *E*_0_, *C*_1_, *C*_2_ and *C*_3_ are the elastic coefficients of the nonlinear elastic spring, *E*_1_ and *θ* are the elastic constant and relaxation time of the Maxwell element, respectively. $\dot{\varepsilon }$ is the strain rate, *θ*_0_ and *β* are related to the material, and ${\dot{\varepsilon }}_{0}$ is the reference strain rate, which was taken to be 1.0 s^−1^ in this paper.

Based on the test data of different strain rates at a certain temperature, the least squares method was used to fit the test curves at different strain rates to minimize the total error of fitting, and to determine the material parameters *E*_0_, *C*_1_, *C*_2_, *C*_3_, and *E*_1_, which are independent of the strain rate, and the relaxation time *θ* related to the strain rate. Combined with Equation (6), all the model parameters can be determined, as shown in Table 2. The R^2^ are 0.969, 0.961, 0.995, 0.998, 0.972, 0.971, respectively.

**Figure 8:**
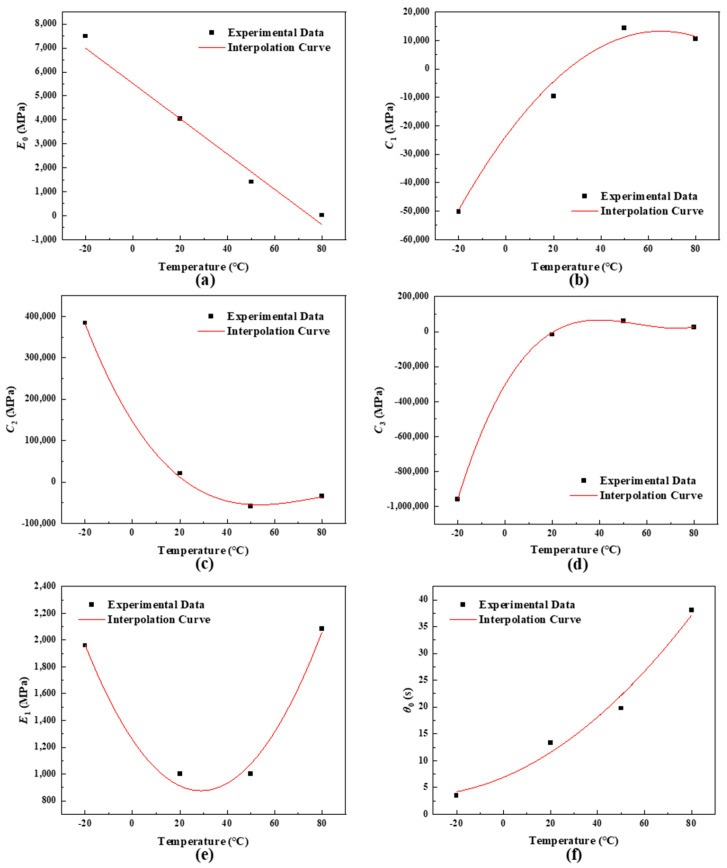
Variation of model parameters with temperature and interpolation curves, and (**a**–**f**) are E0, C1, C2, C3, E1, θ0, respectively.

Obviously, the parameters of the constitutive model except *β* vary greatly with temperature. Therefore, the model parameters are expressed as functions of temperature to study the effect of temperature on these parameters, the expression of the constitutive model is written as follows:

(7) $$\sigma =E_{0}(T)\varepsilon +C_{1}(T)\varepsilon^{2}+C_{2}(T)\varepsilon^{3}+C_{3}(T)\varepsilon^{4}+E_{1}(T)\int_{0}^{t}\dot{\varepsilon }(\tau )\exp\left(-\frac{t-\tau }{\theta }\right)d\tau$$

(8) $$\theta =\theta_{0}(T){\left(\frac{\dot{\varepsilon }}{{\dot{\varepsilon }}_{0}}\right)}^{-\beta }$$

where *T* is temperature in °C, *E*_0_(*T*), *C*_1_(*T*), *C*_2_(*T*), *C*_3_(*T*), *E*_1_(*T*) and *θ*_0_(*T*) are six functions of temperature, which can be determined by interpolation. The model parameters obtained by fitting the experimental data and interpolation curves are shown in Figure 8a–f. It can be seen that the interpolation functions can accurately represent the variation trend of parameters with temperature. The temperature-dependent expressions of the model parameters are determined as follows:

(9) $$\left\{\begin{array}{l} E_{0}(T)=5516.92-73.64T\\ C_{1}(T)=-23490.2+1124.9T-8.60T^{2}\\ C_{2}(T)=145769-9042.25T+127.45T^{2}-0.535T^{3}\\ C_{3}(T)=-302690.2+22731T-443.1T^{2}+2.632T^{3}\\ E_{1}(T)=1255.58-26.25T+0.45363T^{2}\\ \theta_{0}(T)=6.90+0.18376T+0.00243T^{2}\\ \beta =0.25 \end{array}\right.$$

**Figure 9:**
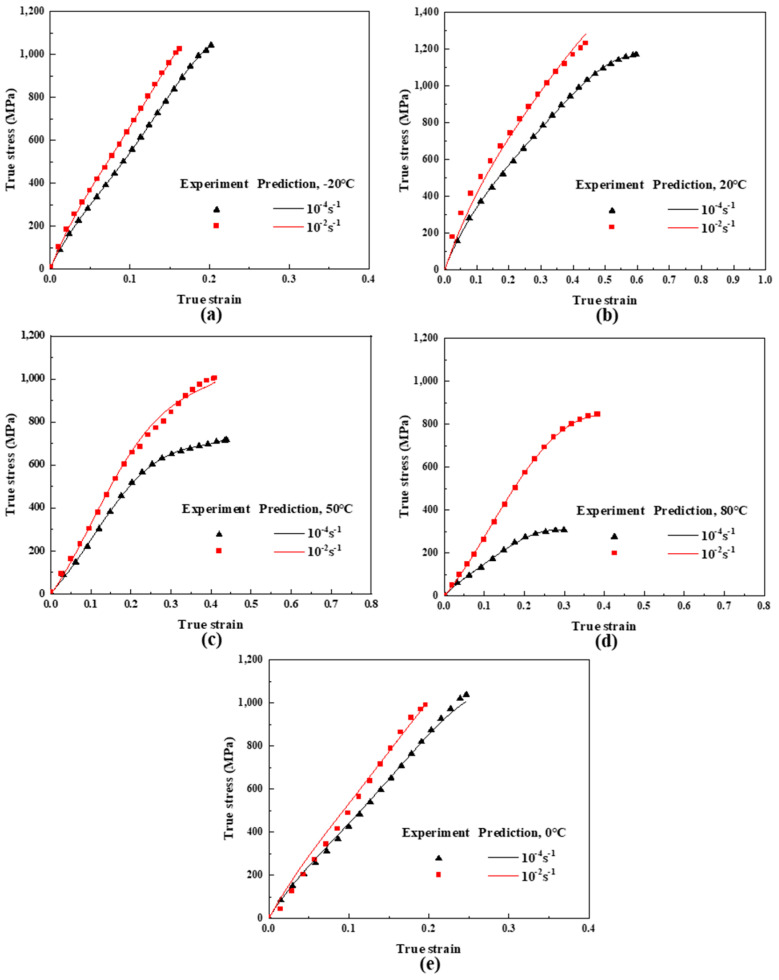
Comparison of stress-strain before yielding of UHMWPE fiber-reinforced composites from the experimental data and the model predictions. The points represent experimental data, and lines represent constitutive model. (**a**) −20 °C; (**b**) 20 °C; (**c**) 50 °C; (**d**) 80 °C; (**e**) 0 °C.

In order to verify the correctness of the constitutive model, a comparison of stress–strain before the yielding of UHMWPE fiber-reinforced composites from the experimental data and the model predictions was made, as shown in Figure 9a–e. The results indicate that the proposed constitutive model can accurately predict the nonlinear viscoelastic response of UHMWPE fiber-reinforced composite materials before yielding at the temperature and strain rate studied in experiments. It is worth noting that the experimental data in Figure 9e (experimental temperature of 0 °C and strain rate of 10^−4^ s^−1^, 10^−2^ s^−1^) were not used to determine the parameters of the constitutive model. By comparing this experimental data with the constitutive model determined in this article, it was found that the constitutive relationship determined in this article can be reliable and correct under quasi-static tension over a large temperature range (−20 °C~80 °C).

## 4. Conclusions

A simple and reliable method was determined and established to carry out uniaxial tensile tests on the materials in the range of −20 °C to 80 °C and at the two strain rates of 10^−4^ s^−1^ and 10^−2^ s^−1^. The experimental results show that the tensile responses of UHMWPE fiber-reinforced composites have significant temperature and strain rate dependence. With the increase in temperature, the yield phenomenon is more obvious, the yield strength and modulus decrease, the fracture strain increases, and the fracture form of the material changes from brittleness to ductility. Both the modulus and yield strength of the material increase significantly with increasing strain rate.

In order to characterize the viscoelastic tensile response of UHMWPE fiber-reinforced composites, a combined phenomenological nonlinear viscoelastic constitutive model was proposed to describe the temperature and strain rate dependent deformation behavior of UHMWPE fiber-reinforced composites before yielding. The parameters of the model were determined by nonlinear fitting and interpolated with a temperature function. In the studied temperature and strain rate range, the comparison of experimental data with model predictions indicates that the proposed model can more accurately predict the nonlinear viscoelastic tensile responses before yielding of UHMWPE fiber-reinforced composites under quasi-static loading over a wide temperature range.

This study presents a meaningful testing technique for the tensile experiments on UHMWPE fiber-reinforced composites. The constitutive relationship of UHMWPE fiber-reinforced composite materials has been well established in a wide temperature range of −20 °C to 80 °C and strain rates of 10^−4^ s^−1^ to 10^−2^ s^−1^.

## Figures and Tables

**Table 1:** Specification parameters of UHMWPE fiber-reinforced composite material without weft.

Material Grade	Fiber Breaking Strength (cN/dtex)	Fiber Initial Modulus (cN/dtex)	Thickness (mm)	Matrix Content	Areal Density (g/m^2^)
AT40H120	≥34	≥1350	0.12	17%	120

**Table 2:** Parameters of the constitutive model at different temperatures.

*T* (°C)	*E*_0_ (MPa)	*C*_1_ (MPa)	*C*_2_ (MPa)	*C*_3_ (MPa)	*E*_1_ (MPa)	*θ*_0_ (s)	*β*
−20	7491.67	−50,180.7	384,223.20	−959,019.00	1959.92	3.43	0.25
20	4036.51	−9683.33	20,755.71	−17,545.00	1002.55	13.28	0.25
50	1407.32	14,444.36	−58,759.32	61,157.00	1001.54	19.75	0.25
80	20.15	10,566.20	−34,916.43	26,079.00	2082.43	38.03	0.25

## Data Availability

The data presented in this study are available on request from the corresponding author. The data are not publicly available due to privacy.
